# Mg_4_Sb_2_O_9_ of the ilmenite structure type

**DOI:** 10.1107/S160053680802789X

**Published:** 2008-09-06

**Authors:** Yuichi Michiue

**Affiliations:** a National Institute for Materials Science, 1-1 Namiki, Tsukuba, Ibaraki 305-0044, Japan

## Abstract

Single crystals of the title compound, tetra­magnesium dianti­monate(V), were obtained by the slow cooling method with K_2_CO_3_. The structure is isotypic with ilmenite, which is constructed by the alternate stacking of layers consisting of metal–oxygen coordination octa­hedra. In each layer, the octa­hedra are connected by sharing edges so as to make holes. One of the two non-equivalent metal sites is fully occupied by Mg  (3 symmetry), while the second metal site (3 symmetry) is disordered and occupied by Mg and Sb with occupation factors of 1/3 and 2/3, respectively.

## Related literature

For ilmenite structures, see: Wechsler & Prewitt (1984 ▶) for FeTiO_3_ and Wechsler & Von Dreele (1989 ▶) for MgTiO_3_. For further phases in the MgO–Sb_2_O_5_ system, see: Kasper (1969 ▶). For related literature, see: Becker & Coppens (1974 ▶); Blasse (1964 ▶); Michiue (2007 ▶).

## Experimental

### 

#### Crystal data

Mg_4_O_9_Sb_2_
*M* *_r_* = 484.7
Trigonal, 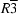

*a* = 5.1722 (11) Å
*c* = 14.028 (2) Å
*V* = 324.99 (11) Å^3^
*Z* = 2
Mo *K*α radiation
μ = 8.73 mm^−1^
*T* = 295 K
0.22 × 0.22 × 0.04 mm

#### Data collection

Rigaku AFC-7R diffractometer
Absorption correction: analytical (*Tompa Analytical*, Rigaku 2004 ▶) *T*_min_ = 0.210, *T*_max_ = 0.671
2359 measured reflections
773 independent reflections
715 reflections with *I* > 2σ(*I*)
*R*_int_ = 0.046
3 standard reflections every 200 reflections intensity decay: 4.6%

#### Refinement

*R*[*F* ^2^ > 2σ(*F* ^2^)] = 0.032
*wR*(*F* ^2^) = 0.074
*S* = 1.55
773 reflections
17 parameters
Δρ_max_ = 3.78 e Å^−3^
Δρ_min_ = −3.64 e Å^−3^

### 

Data collection: *MSC/AFC Diffractometer Control Software* (Molecular Structure Corporation, 1994 ▶); cell refinement: *MSC/AFC Diffractometer Control Software*; data reduction: *CrystalStructure* (Rigaku, 2004 ▶); method used to solve structure: structure of the present compound is isotypic with ilmenite; program(s) used to refine structure: *JANA*2000 (Petříček *et al.*, 2000 ▶); molecular graphics: *ATOMS* (Dowty, 2005 ▶); software used to prepare material for publication: *JANA*2000.

## Supplementary Material

Crystal structure: contains datablocks global, I. DOI: 10.1107/S160053680802789X/si2101sup1.cif

Structure factors: contains datablocks I. DOI: 10.1107/S160053680802789X/si2101Isup2.hkl

Additional supplementary materials:  crystallographic information; 3D view; checkCIF report

## Figures and Tables

**Table 1:** Selected bond lengths (Å), *M* = Mg, Sb

*M*1—O1	2.0527 (15)
*M*1—O1^i^	1.9928 (11)
*M*1—O1^ii^	2.0527 (19)
*M*1—O1^iii^	1.9928 (18)
*M*1—O1^iv^	2.0527 (12)
*M*1—O1^v^	1.9928 (17)
Mg2—O1	2.2091 (17)
Mg2—O1^vi^	2.0455 (17)

Symmetry codes: (i) 
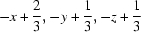
; (ii) 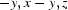; (iii) 
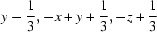
; (iv) 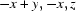; (v) 
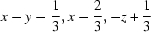
; (vi) 
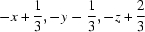
; (vii) 
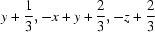
; (viii) 
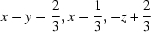
.

**Fig. 1.**
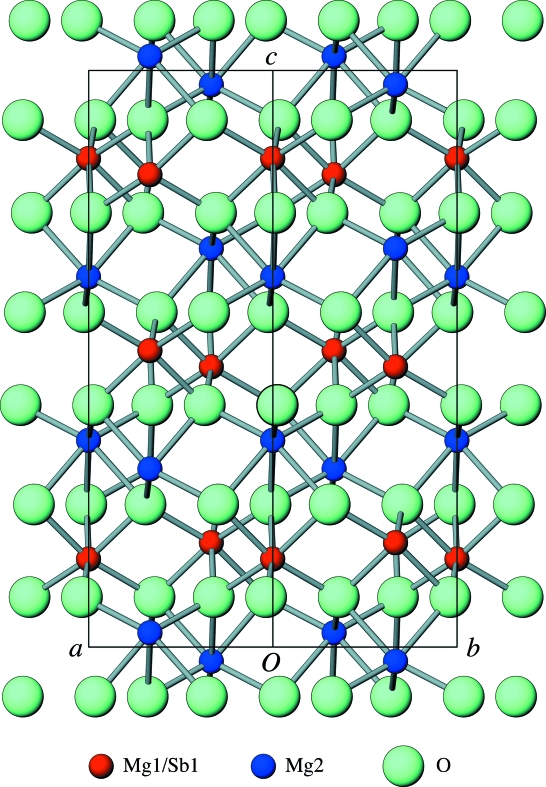
The projection of Mg4Sb2O9 along [110].

**Fig. 2.**
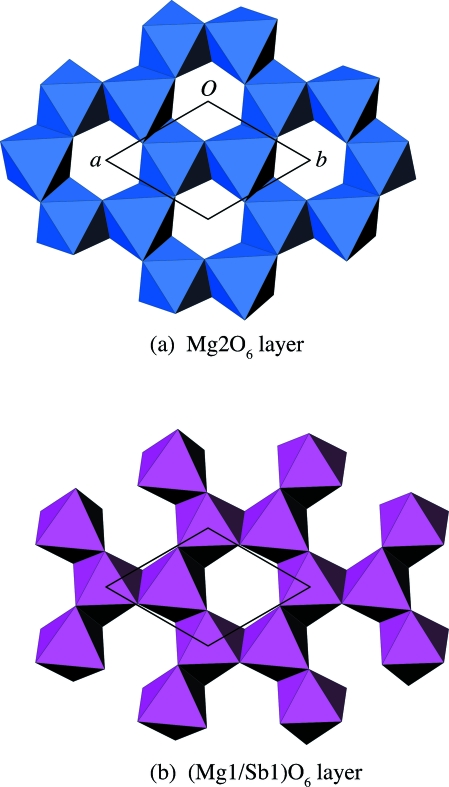
Layers with holes consisting of (a) Mg2O6 octahedra extending around z = 0 and (b) (Mg1/Sb1)O6 octahedra around z = 1/6 in Mg4Sb2O9.

## supplementary crystallographic information

### Comment

A pronounced resemblance of X-ray diffraction patterns for Mg_4_Sb_2_O_9_ and the ilmenite MgTiO_3_ was pointed out by Blasse (1964). In general, ilmenite structure is represented by *A*^2+^*B*^4+^O_3_, that is including equal amounts of divalent and tetravalent cations such as FeTiO_3_ and MgTiO_3_. Although the chemical composition Mg_4_Sb_2_O_9_ is away from that of the typical ilmenite structure, the present analysis has confirmed that the structure of Mg_4_Sb_2_O_9_ is deduced from the ilmenite MgTiO_3_ by replacing the Ti^4+^ ions statistically by 1/3 Mg^2+^ and 2/3 Sb^5+^, as was supposed by Blasse (1964).

The structure is constructed by the alternate stacking of atomic layers along c as shown in Fig. 1. Each of the two nonequivalent metal sites is octahedrally coordinated by six oxygen ions. Two types of layers consisting of edge-shared octahedra are seen in the structure, both of which have holes as illustrated in Fig. 2.

### Experimental

Single crystals of the title compound were obtained unintentionally as the product of a synthesis of K-hollandite by the slow cooling method with excess K_2_CO_3_ (Michiue, 2007).

### Refinement

Partial substitution of Sb for Mg at the Mg2 site was checked by refining the occupation factors of Mg and Sb at the site. In the refinement the full occupation at all the metal and oxygen sites was assumed and the charge neutrality of the whole crystal was kept by imposing constraint conditions. The possibility of the existence of Sb ions at the Mg2 site was excluded because the occupation factor of Sb at the site was slightly negative, -0.001, and that of Mg was 1.001 after the refinement. Thus, it was concluded that Sb ions are only at the Mg1/Sb1 site.

### Crystal data

**Table:**

Mg_4_O_9_Sb_2_	*D*_x_ = 4.952 Mg m^−^^3^
*M**_r_* = 484.7	Mo *K*α radiation, λ = 0.71069 Å
Trigonal, *R*3	Cell parameters from 20 reflections
Hall symbol: -R 3	θ = 9.3–13.5°
*a* = 5.1722 (11) Å	µ = 8.73 mm^−^^1^
*c* = 14.028 (2) Å	*T* = 295 K
*V* = 324.99 (11) Å^3^	Plate, colorless
*Z* = 2	0.22 × 0.22 × 0.04 mm
*F*(000) = 444	

### Data collection

**Table:**

Rigaku AFC-7R diffractometer	715 reflections with *I* > 2σ(*I*)
Radiation source: rotating-anode X-ray tube	*R*_int_ = 0.047
graphite	θ_max_ = 50.1°, θ_min_ = 4.4°
ω/2θ scans	*h* = −11→11
Absorption correction: analytical (Tompa Analytical, Rigaku 2004)	*k* = −11→11
*T*_min_ = 0.210, *T*_max_ = 0.671	*l* = 0→30
2359 measured reflections	3 standard reflections every 200 reflections
773 independent reflections	intensity decay: 4.6%

### Refinement

**Table:**

Refinement on *F*^2^	Primary atom site location: isomorphous structure methods
Least-squares matrix: full	Weighting scheme based on measured s.u.'s *w* = 1/(σ^2^(*I*) + 0.0009*I*^2^)
*R*[*F*^2^ > 2σ(*F*^2^)] = 0.032	(Δ/σ)_max_ = 0.0004
*wR*(*F*^2^) = 0.075	Δρ_max_ = 3.78 e Å^−^^3^
*S* = 1.55	Δρ_min_ = −3.64 e Å^−^^3^
773 reflections	Extinction correction: B-C type 1 Lorentzian isotropic (Becker & Coppens, 1974)
17 parameters	Extinction coefficient: 0.071 (3)
0 restraints	

### Fractional atomic coordinates and isotropic or equivalent isotropic displacement parameters (Å^2^)

**Table:**

	*x*	*y*	*z*	*U*_iso_*/*U*_eq_	Occ. (<1)
Mg1	0	0	0.152618 (14)	0.00628 (7)	0.3333
M1	0	0	0.152618 (14)	0.00628 (7)	0.6667
Mg2	0	0	0.35806 (10)	0.0098 (2)	
O1	0.3091 (3)	0.0125 (3)	0.24710 (7)	0.0078 (3)	

### Atomic displacement parameters (Å^2^)

**Table:**

	*U*^11^	*U*^22^	*U*^33^	*U*^12^	*U*^13^	*U*^23^
Mg1	0.00711 (10)	0.00711 (10)	0.00461 (10)	0.00356 (5)	0	0
M1	0.00711 (10)	0.00711 (10)	0.00461 (10)	0.00356 (5)	0	0
Mg2	0.0080 (3)	0.0080 (3)	0.0133 (4)	0.00401 (13)	0	0
O1	0.0097 (4)	0.0073 (4)	0.0069 (3)	0.0047 (3)	−0.0010 (3)	0.0011 (2)

### Geometric parameters (Å, °)

**Table:**

M1—O1	2.0527 (15)	Mg2—O1	2.2091 (17)
M1—O1^i^	1.9928 (11)	Mg2—O1^vi^	2.0455 (17)
M1—O1^ii^	2.0527 (19)	Mg2—O1^ii^	2.209 (2)
M1—O1^iii^	1.9928 (18)	Mg2—O1^vii^	2.0455 (14)
M1—O1^iv^	2.0527 (12)	Mg2—O1^iv^	2.2091 (15)
M1—O1^v^	1.9928 (17)	Mg2—O1^viii^	2.046 (2)
			
O1—M1—O1^i^	83.77 (5)	O1—Mg2—O1^vi^	87.88 (6)
O1—M1—O1^ii^	82.80 (6)	O1—Mg2—O1^ii^	75.84 (7)
O1—M1—O1^iii^	166.22 (6)	O1—Mg2—O1^vii^	89.32 (5)
O1—M1—O1^iv^	82.80 (6)	O1—Mg2—O1^iv^	75.84 (7)
O1—M1—O1^v^	92.43 (7)	O1—Mg2—O1^viii^	160.12 (8)
O1^i^—M1—O1	83.77 (5)	O1^vi^—Mg2—O1	87.88 (6)
O1^i^—M1—O1^ii^	92.43 (5)	O1^vi^—Mg2—O1^ii^	160.12 (8)
O1^i^—M1—O1^iii^	99.93 (6)	O1^vi^—Mg2—O1^vii^	103.48 (8)
O1^i^—M1—O1^iv^	166.22 (6)	O1^vi^—Mg2—O1^iv^	89.32 (6)
O1^i^—M1—O1^v^	99.93 (6)	O1^vi^—Mg2—O1^viii^	103.48 (7)
O1^ii^—M1—O1	82.80 (6)	O1^ii^—Mg2—O1	75.84 (7)
O1^ii^—M1—O1^i^	92.43 (5)	O1^ii^—Mg2—O1^vi^	160.12 (8)
O1^ii^—M1—O1^iii^	83.77 (7)	O1^ii^—Mg2—O1^vii^	87.88 (6)
O1^ii^—M1—O1^iv^	82.80 (6)	O1^ii^—Mg2—O1^iv^	75.84 (7)
O1^ii^—M1—O1^v^	166.22 (5)	O1^ii^—Mg2—O1^viii^	89.32 (6)
O1^iii^—M1—O1	166.22 (6)	O1^vii^—Mg2—O1	89.32 (5)
O1^iii^—M1—O1^i^	99.93 (6)	O1^vii^—Mg2—O1^vi^	103.48 (8)
O1^iii^—M1—O1^ii^	83.77 (7)	O1^vii^—Mg2—O1^ii^	87.88 (6)
O1^iii^—M1—O1^iv^	92.43 (7)	O1^vii^—Mg2—O1^iv^	160.12 (8)
O1^iii^—M1—O1^v^	99.93 (8)	O1^vii^—Mg2—O1^viii^	103.48 (7)
O1^iv^—M1—O1	82.80 (6)	O1^iv^—Mg2—O1	75.84 (7)
O1^iv^—M1—O1^i^	166.22 (6)	O1^iv^—Mg2—O1^vi^	89.32 (6)
O1^iv^—M1—O1^ii^	82.80 (6)	O1^iv^—Mg2—O1^ii^	75.84 (7)
O1^iv^—M1—O1^iii^	92.43 (7)	O1^iv^—Mg2—O1^vii^	160.12 (8)
O1^iv^—M1—O1^v^	83.77 (6)	O1^iv^—Mg2—O1^viii^	87.88 (6)
O1^v^—M1—O1	92.43 (7)	O1^viii^—Mg2—O1	160.12 (8)
O1^v^—M1—O1^i^	99.93 (6)	O1^viii^—Mg2—O1^vi^	103.48 (7)
O1^v^—M1—O1^ii^	166.22 (5)	O1^viii^—Mg2—O1^ii^	89.32 (6)
O1^v^—M1—O1^iii^	99.93 (8)	O1^viii^—Mg2—O1^vii^	103.48 (7)
O1^v^—M1—O1^iv^	83.77 (6)	O1^viii^—Mg2—O1^iv^	87.88 (6)

Symmetry codes: (i) −*x*+2/3, −*y*+1/3, −*z*+1/3; (ii) −*y*, *x*−*y*, *z*; (iii) *y*−1/3, −*x*+*y*+1/3, −*z*+1/3; (iv) −*x*+*y*, −*x*, *z*; (v) *x*−*y*−1/3, *x*−2/3, −*z*+1/3; (vi) −*x*+1/3, −*y*−1/3, −*z*+2/3; (vii) *y*+1/3, −*x*+*y*+2/3, −*z*+2/3; (viii) *x*−*y*−2/3, *x*−1/3, −*z*+2/3.
